# Malnutrition among patients with Type-2 Diabetes Mellitus

**DOI:** 10.12669/pjms.39.1.5485

**Published:** 2023

**Authors:** Ibrar Ahmed, Hoor Maab Kaifi, Hira Tahir, Adan Javed

**Affiliations:** 1 Ibrar Ahmed, FCPS (Endocrinology), Department of Diabetes and Endocrine, Lady Reading Hospital, Peshawar, Pakistan; 2 Hoor Maab Kaifi, MSc (Foods & Nutrition Sciences), Department of Diabetes and Endocrine, Lady Reading Hospital, Peshawar, Pakistan; 3 Hira Tahir, MSc (Foods & Nutrition Sciences), Department of Diabetes and Endocrine, Lady Reading Hospital, Peshawar, Pakistan; 4 Adan Javed, Pharm D. Department of Diabetes and Endocrine, Lady Reading Hospital, Peshawar, Pakistan

**Keywords:** Malnutrition, Type 2 Diabetes Mellitus, Subjective Global Assessment

## Abstract

**Objectives:**

The present study aims to evaluate the nutritional status of diabetic patients using Subjective Global Assessment (SGA) tool.

**Methods:**

A prospective cross-sectional study was conducted at Endocrine and Medicine Department of Lady Reading Hospital from September 2019 to March 2020. A total of 359 patients diagnosed with Type-2 Diabetes Mellitus (T2DM) were included in the study. The malnutrition status was assessed using SGA, and clinical parameters including albumin and total leukocyte count (TLC).

**Results:**

We have observed mild to moderate malnutrition among 48.2% patients, and severe malnutrition in 10.6% patients as per the SGA scoring. Among the factors associated with nutritional status were BMI (p<0.01), and presence of CAD (DM complication) (p=0.015). As per the correlation analysis, BMI had a significant negative correlation with nutritional status (r=-0.351; p<0.01).

**Conclusion:**

It is concluded from the study results that there is a high prevalence of malnutrition among the enrolled diabetic patients.

## INTRODUCTION

The World Health Organization (WHO) defines malnutrition as shortages or excesses in nutritional intake, an imbalance of vital nutrients, or impaired nutrient utilization resulting in altered body composition.^1^ Malnutrition in hospitalized patients is linked to various unfavorable conditions, including increased infection and complication rates, muscle loss, delayed the healing process, prolonged hospital stay, increased treatment cost, and increased morbidity and mortality.^2^,^3^ The prevalence of malnutrition ranges from 38-52% during the hospital stay, depending upon the patient’s demographic and diagnosis characterstics.^4^ Patients who are elderly and deemed fragile present higher susceptibility for disease-related malnutrition, including decreased protein and energy homeostasis, hormonal abnormalities, and appetite loss.^5^

The critical ICU patients with various disease complaints display changes in the substrate metabolism that leads to nutrient deficiency putting the patient’s body under stress.^6^ Although for diabetes, obesity has been found in the close association among patients, and the treatment mainly focuses on weight loss. However, on the other hand, this obese appearance overshadows other diagnostic features, and the risk of malnutrition remains under-evaluated in the clinical settings.^7^,^8^

Determining what to eat remains the most challenging part of the treatment among diabetic patients. Hence it is essential to identify malnourished patients and those at risk in order to preclude morbidities. However, we have developed knowledge regarding the adverse effects of malnourishment; randomized clinical studies conducted between 2019 and 2016 found that nutritional support decreased mortality and complications while improving functional outcomes and quality of care provided.^9^,^10^ As a result, the European Society for Clinical Nutrition and Metabolism (ESPEN) and the American Society for Parenteral and Enteral Nutrition (ASPEN) have issued practice guidelines that recommend screening for malnutrition, nutritional assessment, and nutritional support for inpatients with malnutrition.^11^,^12^

A patient’s nutritional status is an important indicator of quality care in the institution. Hence the present study aims to evaluate the nutritional status among the diabetic patients presenting at the Endocrine and Medicine Department of Lady Reading Hospital. So that appropriate measures could be taken to improve patient care.

## METHODS

This prospective cross-sectional study was conducted at the Endocrine and Medicine Department of Lady Reading Hospital from September 2019 to March 2020. A sample size of 359 was calculated using WHO sample size calculator determining the sample size in Health Studies, keeping 80% power of the test and 95% confidence interval with a 5% level of significance. All patients (≥ 15 years) diagnosed with T2DM based on the American Diabetes Association (ADA) criteria were recruited via purposive sampling technique. While pregnant females, patients with diabetic ketoacidosis, history of long term intake of drugs that can affect glucose metabolism such as steroids and beta-adrenergic agonists and those with medical/surgical conditions that may affect glucose metabolism, such as acromegaly, Cushing’s syndrome and post-pancreatectomy, were excluded from the study sample.

The socio-demographic and anthropometric data, including age, gender, education, occupation, weight, height, smoking status, BMI, HbA1c, diabetes-associated details etc., were collected using a proforma. The malnutrition was assessed using the SGA tool evaluating weight changes, oral nutritional intake, gastrointestinal symptoms, functional capacity, disease and its relation to nutritional requirements, physical examination and edema/ascites. The patients were classified according to the overall scoring as well-nourished, B mild or moderately-malnourished, and severely-malnourished, which was decided based on the most predominant score (A, B, or C) in the different parts of the SGA. A licensed and trained nutritionist carried out the assessment within 24-72 hours of hospital admission. The level of albumin and total lymphocyte count were also measured for assessment of nutritional risk.

The patient’s baseline demographic and clinical characteristics were presented using mean ± standard deviation, frequency and percentage. An independent sample T-test was used to analyze the association of continuous variables like age, BMI, diabetes duration, HbA1c etc., with nutritional status. Likewise, for categorical variables including gender, diabetes-associated details and nutritional parameters, p-value < 0.05 was considered significant.

Ethical approval was obtained from the Institutional Ethical Review Committee of Lady Reading Hospital (Reference # 216/LRH; Dated 16-09-2019) and written informed consent were obtained from all patients before inclusion.

## RESULTS

The patient’s demographic and clinical characteristics are presented in Table-I. Of the total of 359 diabetic patients enrolled, 57.38% were females. The mean age of the study population was 52.61 ± 11.80 years. Nephropathy was one of the major diabetic complications (22.28%).

**Table-I:** Demographic and clinical characteristics of the enrolled patients.

Baseline characteristics		n=359
Age (years)		52.61±11.80
Weight (kg)		66.30±14.86
Height (cm)		159.56±15.69
BMI (kg/m^2^)		25.30±5.63
HBA1c (%)		10.98±2.46
Duration of DM (years)		11.40±6.61
Gender	Female	206(57.4)
	Male	153(42.1)
BMI Categories	Underweight	20(5.57)
	Normal	76(21.16)
	Overweight	44(12.25)
	Obese	137(38.16)
	Not reported	82(22.8)
Literacy	Illiterate	305(84.95)
	Literate	54(15.04)
Occupation	Working	64(17.82)
	Non-working	295(82.17)
Socioeconomic Status	Average	89(24.79)
	Good	124(34.54)
	Poor	144(40.11)
	Satisfactory	2(0.55)
Smoking Status	Non-smoker	346(96.37)
	Smoker	13(3.62)
Past Medical History	Hypertension	236(65.73)
	Cardiovascular Disease	59(16.43)
	Stroke/Cerebrovascular Disease	27(7.52)
	Foot ulcer	199(55.43)
	Chronic Kidney Disease	73(20.33)
	Dyslipidemia	59(16.43)
DM medications	Both	83(23.11)
	Insulin	239(66.57)
	Oral Hypoglycemic	36(10.02)
	No Medication	1(0.27)
Positive Family history of DM		263(3.25)
DM Complications	Nephropathy	80(22.28)
	Coronary Artery Disease	30(8.35)
	Cerebrovascular Disease	10(2.78)
	Peripheral Vascular Disease	31(8.63)

*All patients had TLC > 1500

The nutritional status was assessed through the SGA tool; 48.2% of the enrolled diabetic patients were mildly/moderately malnourished, 41.2% were well malnourished and 10.6% had severe malnutrition.

The mean BMI significantly declined with the malnutrition status (p<0.01), it was observed that the diabetic patients with normal nutritional status had a mean BMI of 27.32 ± 5.19 kg/m^2^, followed by 24.31 ± 5.09 kg/m^2^ among those with moderate malnutrition while 21.92 ± 6.49 kg/m^2^ among patients with severe malnutrition (Table-II). A significant association was observed between the nutritional status and components of WHO nutritional screening tool (p<0.01). Most of the severely ill cases and those with reduced nutritional intake were mildly/moderately malnourished.

**Table-II:** Baseline characteristics and nutritional status among the diabetic patients in association with nutritional status.

Variable			Nutritional Status (SGA)			p-value

			Well Nourished (n=148)	Mildly/ Moderate Malnourished (n=173)	Severely Malnourished (n=38)	
Baseline characteristics	Age		52.75±10.34	52.21±12.91	53.94±12.06	0.705
	Duration of DM		10.72±6.36	11.83±6.83	12.10±6.42	0.257
	HbA1c		10.85±2.39	11.09±2.54	10.96±2.41	0.682
	BMI		27.32±5.19	24.31±5.09	21.92±6.49	<0.01*
	Gender	Female	88(59.5)	96(55.5)	22(57.9)	0.94
		Male	60(40.5)	77(44.5)	16(42.1)	
	DM medications	Both	34(22.97)	45(26.01)	4(10.5)	0.143
		Insulin	98(66.21)	109(63.0)	32(84.21)	
		No Medication	-	1(0.6)	-	
		Oral Hypoglycemic	16(10.8)	18(10.4)	2(5.3)	
	Family History of DM	No	41(27.7)	46(26.6)	9(23.7)	0.881
		Yes	107(72.3)	127(73.4)	29(76.3)	
	DM Complications	Nephropathy	30(20.3)	40(23.1)	10(26.3)	0.679
		CAD	7(4.7)	22(12.7)	1(2.6)	0.015*
		CVA	4(2.7)	5(2.9)	1(2.6)	0.993
		PVD	14(9.5)	14(8.1)	3(7.9)	0.897
WHO nutritional screening	BMI	> 20 (>30 Obese)	98(90.7)	88(75.9)	15(48.4)	<0.01*
		18.5 - 20	8(7.4)	20(17.2)	6(19.4)	
		< 18.5	2(1.9)	8(6.9)	10(32.3)	
	Unplanned	Yes	102(68.9)	148(85.5)	37(97.4)	<0.01*
	weight loss in					
	past 3-6 months	No	46(31.1)	25(14.5)	1(2.6)	
	Reduced nutritional intake	Yes	59(39.9)	109(63.0)	30(78.9)	<0.01*
		No	89(60.1)	64(37.0)	8(21.1)	
	Severely ill	Yes	117(79.1)	154(89.0)	36(94.7)	0.010*
		No	31(20.9)	19(11.0)	2(5.3)	

* p<0.05 is considered significant.

The total leukocyte count and albumin were also estimated; it was found that the TLC was > 1500 among all the enrolled T2DM patients. Furthermore, the observed albumin levels were similar among the moderate and severely malnourished groups; only 6(3.5%) and 4(10.5%) patients of the respective groups had albumin levels < 2.5 g/dl, respectively.

Table-III shows the correlation analysis; BMI had a significant negative correlation with nutritional status (r= - 0.351; p<0.01). While albumin had positive significant correlation with SGA scoring (r=0.232; p<0.05).

**Table-III:** Correlations between clinical parameters and nutritional status.

	Age	BMI	Albumin	HBA1c	Diabetes Duration	SGA
Age	1.000	0.116	0.035	-0.097	0.175**	0.023
BMI		1.000	-0.243**	-0.151*	0.060	-0.351**
Albumin			1.000	-0.052	0.144^**^	0.232^**^
HBA1c				1.000	-0.017	0.040
Diabetes Duration					1.000	0.070
SGA						1.000

** Correlation is significant at the 0.01 level (2-tailed).

* Correlation is significant at the 0.05 level (2-tailed).

## DISCUSSION

Although systematic nutritional screening is becoming common in the healthcare sector with advancements in medicine and clinical care, in-hospital malnutrition remains prevalent.^13^ It is not considered a medical priority and is often overlooked. Several studies and scientific societies recommend nutritional evaluation among hospitalized patients upon admission, as it reduces the overall healthcare costs and also improves the disease outcome.^14^,^15^ The current study revealed severely malnourishment among 10.6% of the enrolled diabetic patients, 41.2% were well nourished while 48.2% were mildly/moderately malnourished as defined by SGA tool. In contrast, Carolyn et al. observed a comparatively higher risk of malnutrition among diabetic patients.^16^ Although malnutrition related diabetes mellitus is commonly reported in India, Bangladesh, Sri Lanka, and many other countries^17^,^18^ but it was relatively less common in Pakistani Population as suggested by local case study from Karachi by Naim et al.^19^ Furthermore, another study determining the malnutrition risk among hospitalized patients with various chronic conditions suggested that there is a comparatively high risk of malnutrition among the diabetic, renal patients and those with prolonged hospital stay (p<0.05).^20^

Several epidemiological studies have presented a significant relationship between increased BMI and T2DM risk among Asian population.^21^-^24^ Significant variation in the patient’s BMI with nutritional status was observed (p<0.01). The SGA rating showed that the mild/moderately and severely malnourished patients had low BMI as compared to the well-nourished patients. In support, a study also reported moderate malnutrition among obese class-3 and overweight patients while severe malnutrition reported among 83% of underweight patients.^16^ Furthermore, a higher level of protein depletion and low TLC has been documented among patients with high nutritional risk.^20^,^25^,^26^ Surprisingly our findings were contradictory concerning both of these clinical parameters, i.e., most of the diabetic patients with moderate (73.4%) and severe (44.7%) malnutrition had albumin levels > 3.4 g/dl. In contrast, studies reveal significant correlation between malnutrition and serum albumin (depletion) among elderly patients with diabetes.^27^,^28^

As per the correlation analysis, BMI had a significant negative correlation with malnutrition status (r=-0.351; p<0.01). Age had a strong positive significant correlation with duration of diabetes (r=0.175; p<0.01) and a positive non-significant correlation with nutritional status (r=0.023). Also, the significant positive correlation between albumin and nutritional status (SGA) was quite surprising (r=0.232; p<0.05). The role of serum biomarkers, specifically albumin, in diagnosing or monitoring malnutrition remains controversial due to the lack of associated specificity and longer half-life (approx. 20 days).^29^

### Limitations:

Despite having sufficient sample size for the detection of malnutrition and sub-group analysis, the study had certain limitations that need to be disclosed. The major limitation was the cross sectional design and single center approach of the study, which limited the generalizability of the results.

## CONCLUSION

Malnutrition is prevalent among hospitalized T2DM patients. Earlier diagnosis and provision of nutritional intervention among diabetic patients not only help reduce the length of hospital stay but also speed up the recovery process and prevents DM complications. Therefore, the present study emphasized the importance of assessing the nutritional status among admitted T2DM as it helps in the identification of malnourished patients and those at risk to preclude morbidities. Future studies with large sample size, assessing nutritional status with different tools and at multiple time points (follow-ups) are recommended to develop nutrition interventions and strategies for the appropriate management of patients with chronic illnesses.

## References

[1] WHO (2021). Malnutrition Factsheet.

[2] Marin S, Serra?Prat M, Ortega O, Audouard Fericgla M, Valls J, Palomera E (2021). Healthcare costs of post?stroke oropharyngeal dysphagia and its complications:malnutrition and respiratory infections. Eur J Neurol.

[3] Younis H, Younis S, Ahmad S (2019). Awareness regarding complications of type II diabetes mellitus among diabetics in Karachi, Pakistan. Int J Endorsing Health Sci Res.

[4] Correia MI, Sulo S, Brunton C, Sulz I, Rodriguez D, Gomez G (2021). Prevalence of malnutrition risk and its association with mortality:nutrition Day Latin America survey results. Clin Nutr.

[5] Cederholm T, Barazzoni RO, Austin P, Ballmer P, Biolo GI, Bischoff SC (2017). ESPEN guidelines on definitions and terminology of clinical nutrition. Clin Nutr.

[6] Mehanna H, Nankivell PC, Moledina J, Travis J (2009). Refeeding syndrome–awareness, prevention and management. Head Neck Oncol.

[7] Klinkenberg M, Thijs A (2005). Profile of the malnourished patient. Eur. J. Clin. Nutr.

[8] Yildirim Z, Uzunlulu M, Caklili O, Mutlu H, Oğuz A (2018). Malnutrition rate among hospitalized patients with type 2 diabetes mellitus. Prog. Nutr.

[9] Bally MR, Yildirim PZ, Bounoure L, Gloy VL, Mueller B, Briel M (2016). nutritional support and outcomes in malnourished medical inpatients:a systematic review and meta-analysis. JAMA Intern Med.

[10] Schuetz P, Fehr R, Baechli V, Geiser M, Deiss M, Gomes F (2019). Individualised nutritional support in medical inpatients at nutritional risk:a randomised clinical trial. Lancet.

[11] Kaegi-Braun N, Mueller M, Schuetz P, Mueller B, Kutz A (2021). Evaluation of nutritional support and in-hospital mortality in patients with malnutrition. JAMA Netw Open.

[12] Gomes F, Schuetz P, Bounoure L, Austin P, Ballesteros-Pomar M, Cederholm T (2018). ESPEN guidelines on nutritional support for polymorbid internal medicine patients. Clin Nutr.

[13] Farooq MU, Mushtaq F, Naeem Z, Iqbal S, Naseem S, Ishtiaq O (2018). Dietary habits and practices of type-2 diabetic patients in a tertiary care centre of Islamabad, Pakistan. J Pak Med Assoc.

[14] lvarez HJ, Planas VM, Leon SM, Garcia DA, Celaya PS, Garcia LP (2012). Prevalence and costs of malnutrition in hospitalized patients;the PREDYCES Study. Nutr Hosp.

[15] A.S.P.E.N. Board of Directors and the Clinical Guidelines Task force (2002). Guidelines for the use of parenteral and enteral nutrition in adult and pediatric patients J Parenter Enteral Nutr.

[16] Carolyn NM, Leonora DR, Luisa CC (2016). Prevalence of malnutrition among patients with diabetes mellitus type 2 admitted in a tertiary hospital. Philipp J Intern Med.

[17] Bajaj JS, Krall LP Malnutrition related ketosis-resistant diabetes mellitus-Classification, causes and mechanism. World book of diabetes in practice, Krall LP. (ed).

[18] Geevarghese P (1970). Comparison of study of pancreatic diabetes mellitus and maturity onset diabetes mellitus. J Ind Med Assoc.

[19] Naim M, Shera AS (1993). Malnutrition-Related Diabetes Mellitus (MRDM)-An Uncommon Disorder in Pakistan. J Pak Med Assoc.

[20] Behiry ME, Salem MR (2019). High prevalence of malnutrition among hospitalized patients in a tertiary care hospital by using malnutrition universal screening tool. Egypt J Intern Med.

[21] Araneta MR, Kanaya AM, Hsu WC, Chang HK, Grandinetti A, Boyko EJ (2015). Optimum BMI cut points to screen Asian Americans for type 2 diabetes. Diabetes Care.

[22] McNeely MJ, Boyko EJ (2004). Type 2 diabetes prevalence in Asian Americans:results of a national health survey. Diabetes Care.

[23] Ismail A, Fatima F (2018). Maternal nutritional knowledge and its association with iron deficiency anemia in children. Int J Endorsing Health Sci Res.

[24] Hazrat H, Ahmed S, Noushad S (2018). The association of maternal age, body mass index, physical activity with Gestational Diabetes Mellitus. Int J Endorsing Health Sci Res.

[25] Paris AS, Garcia JM, Candela CG, Burgos R, Martin A, Matia P (2013). Malnutrition prevalence in hospitalized elderly diabetic patients. Nutr Hosp.

[26] Kong DX, Xiao YX, Zhang ZX, Liu YB (2020). Study on the Correlation between Metabolism, Insulin Sensitivity and progressive weight loss change in Type-2 Diabetes. Pak J Med Sci.

[27] Gomez Ramos MJ, Gonzalez Valverde FM, Sanchez Alvarez C (2005). Nutritional status of a hospitalised aged population. Nutr Hosp.

[28] Vischer UM, Frangos E, Graf C, Gold G, Weiss L, Herrmann FR (2012). The prognostic significance of malnutrition as assessed by the Mini Nutritional Assessment (MNA) in older hospitalized patients with a heavy disease burden. Clin Nutr.

[29] Levitt DG, Levitt MD (2016). Human serum albumin homeostasis:a new look at the roles of synthesis, catabolism, renal and gastrointestinal excretion, and the clinical value of serum albumin measurements. Int J Gen Med.

